# Enhancement of Terahertz Emission by Silver Nanoparticles in a Liquid Medium

**DOI:** 10.3390/mi14081593

**Published:** 2023-08-13

**Authors:** Haoyang Wang, Tao Shen, Jinkun Liu, Yan Zhu, Hong Li, Tianwu Wang

**Affiliations:** 1 Faculty of Information Engineering and Automation, Kunming University of Science and Technology, Kunming 650500, China; 2 Faculty of Materials Science and Engineering, Kunming University of Science and Technology, Kunming 650500, China; 3 GBA Branch of Aerospace Information Research Institute, Chinese Academy of Sciences, Guangzhou 510700, China

**Keywords:** terahertz emission, femtosecond laser pulses, silver nanoparticles, liquid terahertz sources

## Abstract

Due to higher molecular density, lower ionization potential, and a better self-healing property compared with gases, liquid targets have been used for laser-induced terahertz generation for many years. In this work, a liquid target used for terahertz radiation is embedded with silver nanoparticles (Ag NPs), which makes the material have both the fluidity of liquids and conductivity of metals. Meanwhile, the experimental setup is easier to implement than that of liquid metals. Polyvinyl alcohol (PVA) is used as a stabilizing agent to avoid precipitation formation. It is observed that the power of 0.5 THz radiation from the Ag NP suspension is five times stronger than that from liquid water in identical experimental conditions. In addition, the reusability of the material is investigated using multiple excitations. UV–visible spectroscopy and TEM imaging are carried out to analyze the target material after each excitation. As a result, quasispherical Ag NP suspensions show good reusability for several excitations and only a decrease in particle concentration is observed. By contrast, the chain-like Ag NP suspension shows poor stability due to PVA damage caused by intense laser pulses, so it cannot be used in a recyclable manner.

## 1. Introduction

Laser-induced air plasma has been one of the most important terahertz sources in the last two decades, since it is cost-effective, simple in its structure, and does not cause permanent damage [1,2,3,4,5]. Liquids have a higher molecular density, lower ionization potential, and better self-healing properties compared with gases, so liquids have been demonstrated as a preferable medium for terahertz emission [6,7,8,9,10]. Since the first experiment of terahertz radiation from liquid water was performed [11,12], many approaches have been proposed to optimize experimental systems and boost terahertz radiation, such as multicolor excitation [13], double-pump excitation [14,15], tailoring laser beams [16], and so on. The multicolor scheme increases the asymmetry between the front and the back edges of laser pulses, resulting in a stronger transient photocurrent [17,18]. The double-pump scheme increases terahertz emission via preformed plasma created by the first pump [14]. As for the optimization of target materials, many kinds of liquids have been explored, such as heavy water, acetone, α-Pinene, ethanol, and liquid nitrogen [19,20,21,22]. However, as a transparent target, terahertz radiation from a liquid saturates with the laser intensity because of the intensity clamping effect [23].

In recent years, liquid metal has been recognized as a strong medium for terahertz radiation. There are several reasons accounting for this: (1) metals have a relatively lower ionization threshold, indicating that more carriers can be created under the same laser excitation; (2) metals have free electrons both originally existing in it and excited by laser pulses; (3) metals have a higher surface tension, which leads to a smoother surface [24]. Liquid gallium was first demonstrated as a liquid metal source for terahertz wave generation because of its chemical stability, physical safety, and accessibility [25]. The melting point of gallium is 30 °C. Therefore, a high-quality liquid gallium line can be obtained using a heater-attached liquid circulating system [24]. As a result, terahertz radiation with a stronger electric field and broader bandwidth is obtained from liquid gallium compared to that from liquid water under identical optical conditions [26]. The radiation from liquid gallium is attributed to coherent transition radiation [27,28]. The free electrons in the liquid metal are accelerated by a ponderomotive force, which results from the inhomogeneous distribution of laser intensity, and a terahertz wave is emitted when the electrons cross the boundary of gallium and air [26].

The eutectic alloy of tin and indium (48% Sn and 52% In) has been proposed to generate terahertz waves as well [29]. In the cited experiment, the metal was in the liquid state due to a preheating process at 140 °C. With the help of a piezoelectric actuator bonded to the glass capillary of a dispenser and a CCD camera, laser pulses and droplets could be synchronized. An intense signal was observed when the alloy was excited by two pulses delayed relative to each other in time. By adjusting the delay between them, the intensity of terahertz radiation increased by more than three orders of magnitude. Terahertz radiation from this alloy is explained by the result of the longitudinal distribution of electron density under the action of the ponderomotive force caused by the second laser pulse [29].

The setup of the liquid metal experiment mentioned above is rather complex, and many requirements need to be fulfilled. In addition, it is difficult to select an appropriate material. It is supposed to be in a liquid state within an environment which can be created in the laboratory. However, many factors should be taken into consideration, such as the melting point, surface tension, density, viscosity, ionization energy, chemical stability, and physical safety [24]. In order to make more kinds of metals available for terahertz radiation and simplify the experimental system, a strategy that embeds solid metal particles into a liquid medium was employed in this work. According to previous work, the generation process takes place only at the surface of a metal target because of the much thinner penetration depths in metal of both lasers and terahertz waves [25]. Therefore, metal particles of a nanometer scale were adopted. Well-dispersed nanoparticles introduce more surfaces compared to liquid metal when the quantity of metal is the same, so a higher utilization of metal material can be achieved.

Silver nanoparticles (Ag NPs) have antibacterial properties, so they have been of great interest both in research projects and practical applications in recent decades [30,31]. However, silver has good conductivity, indicating that abundant charge carriers can be utilized for terahertz generation. In addition, a sophisticated synthesis process of Ag NPs has been available for many years. Therefore, liquid water which was embedded with Ag NPs was used as a target for terahertz generation in this work. Polyvinyl alcohol (PVA) was used as a stabilizing agent to avoid precipitation formation, and thus, well-dispersed Ag NP suspensions could be made. Since liquid water was used as the solvent, the experiment result of Ag NP suspensions can be compared with that of pure water, for which there are many available published results from previous years, helping us understand the underlying mechanism of terahertz radiation from liquids and metals.

In this work, an enhancement in terahertz radiation at 0.5 THz from liquid water with Ag NPs was demonstrated experimentally. As a result, the radiant power from Ag NP suspensions was five times stronger than that from liquid water in identical experimental conditions. In terms of liquid-based terahertz sources, the reusability of liquids greatly reduces costs and makes experimental systems environmentally friendly. Hence, the reusability of the material was investigated using multiple excitations, and methods of analysis, such as UV–visible spectroscopy and TEM imaging, were performed on the target material after each excitation.

## 2. Materials and Methods

In this section, the material and experimental setup in this study are introduced. First, the preparation process of Ag NP suspensions is described. Then, the prepared material is analyzed by Transmission Electron Microscopy (TEM), Energy-Dispersive X-ray spectroscopy (EDX), and UV–visible spectroscopy. Finally, the experimental setup for terahertz radiation in this work is presented.

### 2.1. The Synthesis of Ag NP Suspensions

**Figure 1:**
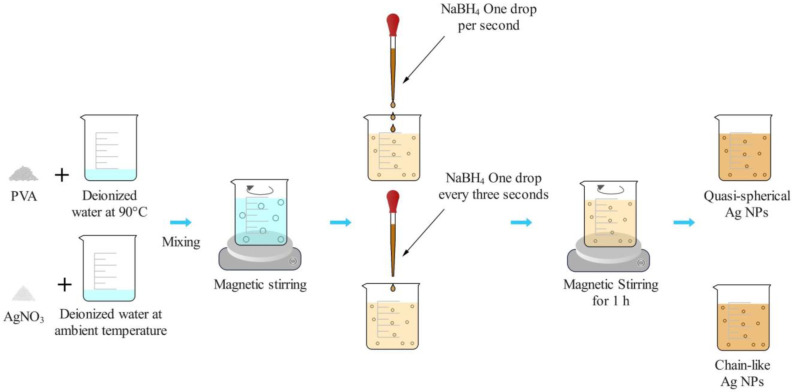
Schematic diagram of Ag NP preparation process.

**Figure 2:**
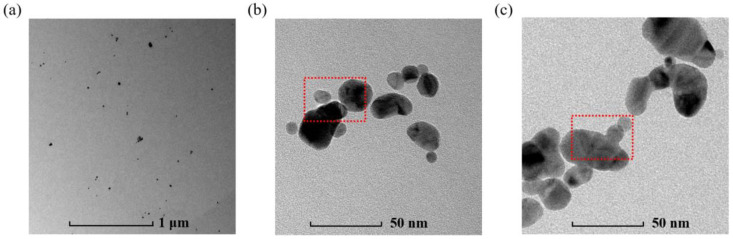
(**a**) TEM image of well-dispersed Ag NPs in the medium. (**b**) Detailed image of quasispherical Ag NPs. The particles in the red dashed box have clear edges and do not connect to the surrounding particles. (**c**) Detailed image of chain-like Ag NPs. The particles in the red dashed box are connected to each other and further linked into chains.

The chemical reduction method was adopted to prepare the Ag NP suspensions, which used AgNO_3_ as the Ag source, NaBH_4_ as the reductant, deionized water as the solvent, and PVA as the surfactant. PVA protects Ag NPs from aggregation and establishes a good dispersion in aqueous media [32,33]. To prevent the synthesized Ag NPs from agglomerating in the liquid, the solutions of AgNO_3_ and PVA were mixed first, and the reduction of Ag^+^ ions was carried out by adding the NaBH_4_ solution dropwise into the mixed solution. In fact, the dropping rate of the NaBH_4_ solution has an influence on the nanostructure of Ag NPs. If the drops are frequent, such as one drop per second, one tends to obtain well-dispersed quasispherical Ag NPs without aggregation, but if the dropping rate of NaBH_4_ is low, such as one drop every three seconds, the Ag NPs will aggregate together to form a chain-like structure. The detailed preparation process is summarized in a schematic diagram in Figure 1. Through this method, the final Ag NP suspensions with the desired concentrations (ppm) were successfully prepared. Figure 2 shows the TEM image of the prepared material. From Figure 2a, we can observe that the Ag NPs were well dispersed in the liquid media. Figure 2b is a detailed TEM image of the quasispherical Ag NPs made by adding the NaBH4 solution at one drop per second. Although the Ag NPs in Figure 2b are close to each other, each of their edges is clear and does not connect to the surrounding particles (as shown in the red box), indicating that no Ag agglomeration happened, while the nanoparticles in Figure 2c are connected to each other and further linked into chains (as shown in the red box); because of this characteristic, they were named chain-like Ag NPs.

### 2.2. Characterizations of Ag NP Suspensions

**Figure 3:**
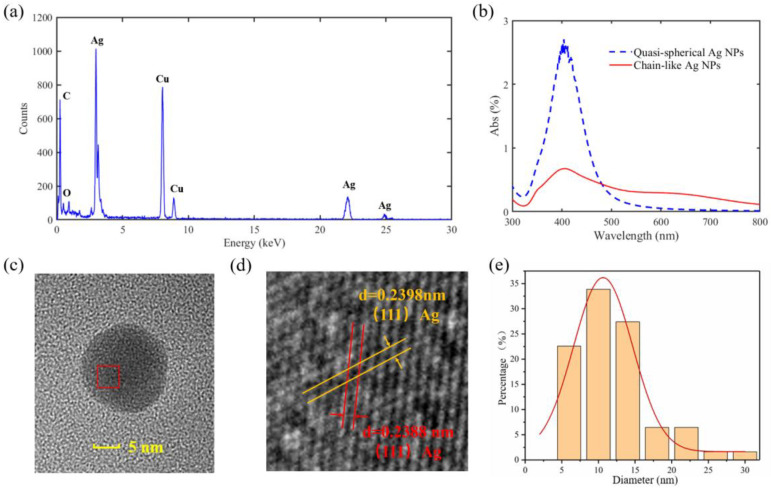
(**a**) EDX picture of the Ag NP suspension; (**b**) UV–visible spectroscopy of the Ag NP suspension with quasispherical and chain-like structure; (**c**) TEM image of a single Ag NP; (**d**) close-up of the red box in Figure 3c with the interplanar distance labeled; (**e**) statistical graph of particle diameter distribution of Ag NPs. The orange box shows the percentage of particles within certain diameter range and the red line is a Gaussian fit to the distribution.

In order to investigate the morphological characteristics of the Ag NPs in the suspensions, several characterizations were adopted to analyze the prepared material. First, EDX was carried out to specify the composition of the Ag NP products, as shown in Figure 3a. Except for silver, there were other elements like carbon, oxygen, and copper in them. Carbon and oxygen came from the short chains after the hydrolysis of PVA, while copper was derived from the copper mesh used for TEM observation. In addition, UV–visible spectroscopy was performed for determining whether Ag NPs existed in the suspensions or not, because Ag NPs perform surface plasmon absorption between 400 nm and 450 nm [34]. As shown in Figure 3b, the results of the UV–visible spectrum reveal plasmon absorption peaks near 404 nm for both the quasispherical and the chain-like Ag NPs, indicating that the reduction of Ag NPs indeed proceeded in the liquid. Figure 3c shows a TEM image of a single Ag NP with a diameter of ~15 nm. The red box is labeled to determine the interplanar crystal spacing, and the corresponding result is displayed in Figure 3d. The calculated values for the interplanar spacing were 0.2398 and 0.2388 nm, and both were basically in accordance with the theoretical value of the (111) planes of Ag, indicating that the (111) plane was exposed on the surfaces of the Ag NPs. In addition, a statistic graph of the nanoparticle diameter is presented in Figure 3e. The average diameter of the Ag NPs was ~12 nm.

### 2.3. Experimental Setup

**Figure 4:**
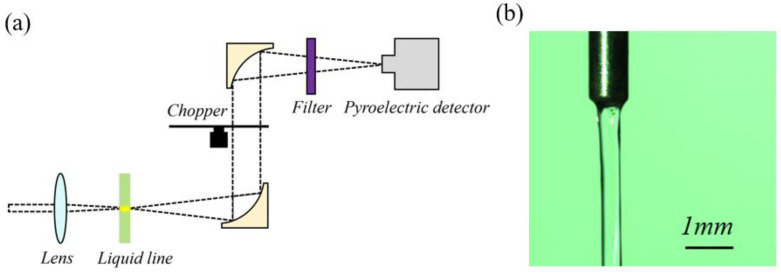
(**a**) Experimental setup of terahertz emission from Ag NP suspensions induced by femtosecond laser pulses; (**b**) close-up picture of the liquid flow.

The experimental setup of terahertz radiation from the Ag NP suspension is presented in Figure 4a. We used a Spectra Physics Spitfire Ti: sapphire regenerative amplifier system that delivers optical pump pulses of 35 fs duration, of 800 nm central wavelength, and at a repetition rate of 1 kHz. The beam diameter (1/e^2^) was 10 mm and the average power of the laser pulses used in this experiment ranged from 0.2 to 3.0 W. A Gaussian pulse shape (0.7 deconvolution factor) was used to determine the pulse width (FWHM) from an autocorrelation signal. The power of terahertz radiation from the liquid was measured with a Gentec-EO T-RAD pyroelectric detector. Each value was sampled 75 times and the mean values with standard errors are presented in the experimental results section. Femtosecond laser pulses were focused into the liquid line using a convex lens with a focal length of 50.8 mm. Terahertz waves radiated by the liquid line induced by laser pulses were collected in the pyroelectric detector via a pair of off-axis parabolic mirrors. In order to avoid damage to the detector, a high-resistivity silicon wafer was used as a filter to block the residual laser beams. Since 0.5 THz is the central frequency of most ultrafast terahertz systems [35], the radiation centered at 0.5 THz was measured in this experiment. The green line in this figure represents the flowing liquid line of the Ag NP suspensions, which was formed using a combination of a syringe and a stepping motor. The syringe needle had a diameter of 600 µm and the liquid had a flow rate of 20 mL/min. By adjusting the flow rate of the liquid and diameter of the syringe needle, a quasilaminar flow was achievable. Figure 4b is a close-up picture of the liquid flow, which provides a pristine smooth interface, indicating the energy loss of the laser and terahertz radiation caused by the air–liquid interface could be reduced.

## 3. Results

In this section, the experimental results of this study obtained using the experimental setup and materials mentioned above are elaborated. First, the terahertz radiation from the Ag NP suspension was measured and compared with liquid water. Furthermore, the reuse of these materials was investigated by testing them in several consecutive excitations. Since the liquids with quasispherical Ag NPs and chain-like Ag NPs showed different radiation properties, this section is divided into two parts.

### 3.1. Quasispherical Ag NPs

**Figure 5:**
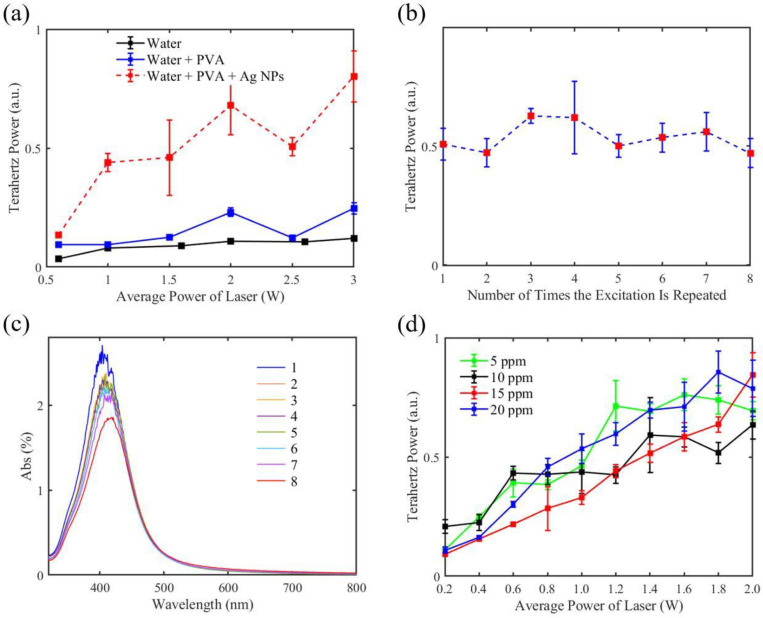
(**a**) Experimental results of terahertz emission induced from water, the PVA solution, and the PVA-stabilized Ag NP suspension when the average power of femtosecond laser pulses increased. (**b**) Measured power of terahertz radiation with increasing number of excitations. (**c**) UV–visible spectroscopy of the PVA-stabilized Ag NP suspension after each excitation. The number indicates the number of times the excitation is repeated. (**d**) Measured power of terahertz radiation with increasing average power of femtosecond laser pulses when the NP concentration is 5 ppm, 10 ppm, 15 ppm, and 20 ppm, respectively.

The terahertz emission from the liquid with quasispherical Ag NPs induced by femtosecond laser pulses is shown in Figure 5a. It is noteworthy that the terahertz radiation from the PVA-stabilized Ag NP suspensions (shown by the red dotted line) was much stronger than that from pure liquid water and water with PVA. The radiant power of the terahertz wave at 0.5 THz from the liquid with Ag NPs was approximately five times stronger than that from the liquid water in this experiment. Pure water showed the weakest radiation. When the PVA was added to the liquid water, the terahertz radiation from it was slightly enhanced, indicating that the primary factor of the radiation enhancement was not the existence of the PVA but the Ag NPs.

In subsequent experiments, the liquid with quasispherical Ag NPs was excited by femtosecond laser pulses eight times and the terahertz radiation each time was measured, as shown in Figure 5b. It can be observed that the radiation power each time did not change significantly. UV–visible spectroscopy was carried out to investigate the morphologies of the medium after each excitation, as shown in Figure 5c. The results reveal that the absorption peak of the medium diminished when the number of excitations increased. According to the Beer–Lambert law, the absorption peak of UV–visible spectroscopy is a linear function of Ag NP concentration at room temperature [36], so Figure 5c implies a small decrease in NP concentration in the range of 0~20 ppm. In order to investigate the relationship between the radiation ability of the Ag NP suspension and its NP concentration, we measured the terahertz radiation from the quasispherical Ag NPs with different NP concentrations below 20 ppm, as shown in Figure 5d. It was observed that the terahertz radiation showed an upward trend as the laser power increased, but no direct dependence between the NP concentration and terahertz radiation was established. It can be inferred that the distinction between the experimental results is beyond the measurement accuracy of this experiment. However, to some extent, we can draw the conclusion that when the NP concentration is at a low level (<20 ppm), it has little effect on the power of terahertz emission induced by femtosecond laser pulses.

**Figure 6:**
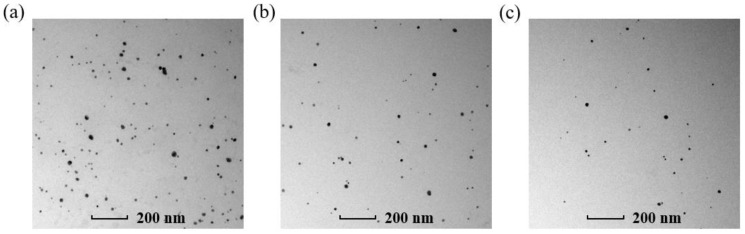
TEM image of the quasispherical Ag NP suspension after (**a**) fourth, (**b**) sixth, and (**c**) eighth excitations, respectively.

Finally, it was demonstrated by the TEM image that the Ag NP concentration decreased when the material was excited by femtosecond laser pulses. Figure 6 shows TEM images of the Ag NP suspensions after the fourth, sixth, and eighth laser excitations. It can be observed that the number of particles in the liquid reduced when the number of excitations increased, which is consistent with the conclusion of concentration decrease. It should be noted that the absorption peaks in Figure 5c show a redshift as the number of excitations increases. We attribute this phenomenon to the aggregations and coalescences of Ag NPs caused by laser excitation.

### 3.2. Chain-Like Ag NPs

**Figure 7:**
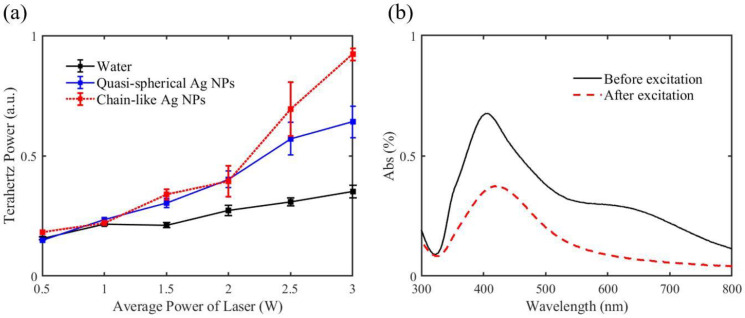
(**a**) Experimental results of terahertz emission from liquid water, the quasispherical Ag NP suspension, and the chain-like Ag NP suspension when the average power of laser pulses increased; (**b**) UV–visible spectroscopy of the chain-like Ag NP suspension before and after laser excitation, respectively.

**Figure 8:**
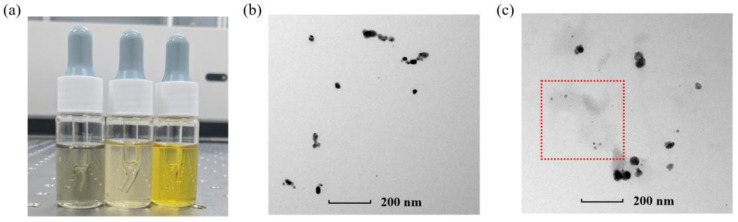
(**a**) Picture of chain-like Ag NP suspension before excitation, chain-like Ag NP suspension after excitation, and quasispherical Ag NP suspension, respectively; (**b**) TEM image of chain-like Ag NP suspension before excitation; (**c**) TEM image of chain-like Ag NP suspension after excitation. Damaged PVA caused by laser pulses is shown in red box.

The experimental results of the Ag NP suspensions with a chain-like nanostructure are shown in Figure 7a. It can be observed that the chain-like Ag NPs emitted more intense terahertz radiation than the quasispherical Ag NPs when under the same laser pulses. Furthermore, this higher yield increased with increasing laser power. Although the liquid with chain-like Ag NPs exhibited a better capacity for terahertz radiation, it was unstable when excited by the laser pulses. Figure 7b shows the UV–visible spectroscopy of the medium before and after laser excitation. According to the figure, the first peak of the chain-like Ag NPs drops dramatically and the second peak disappears when the medium is excited by laser pulses, indicating the morphology of the chain-like Ag NPs changed significantly after the laser excitation. In fact, the difference between them can be seen directly with the naked eye. As shown in Figure 8a, the liquid with chain-like Ag NPs changed its color from black to pale yellow after laser excitation. Therefore, a TEM image of the medium before and after excitation was recorded, as shown in Figure 8b,c, respectively. It can be observed that the medium before excitation was in a clear view, indicating the Ag NPs were dispersed in a well-functioning PVA solution, while the liquid after excitation exhibited shades of gray (as shown in the red box), which were recognized as the damaged PVA. Therefore, it is inferred that the instability of the liquid with chain-like Ag NPs came from the damaged PVA caused by intense femtosecond laser pulses.

## 4. Discussion

Compared with liquid water, an enhancement in terahertz emission from Ag NP suspensions induced by femtosecond laser pulses was demonstrated experimentally. The mechanism of terahertz radiation from liquid water has been explained in previous works. At the beginning, electrons in the liquid water are ionized by the intense laser pulses. Then, the electrons are accelerated by a ponderomotive force caused by the inhomogeneous distribution of laser intensity. Compared to electrons, ions are many orders of magnitude heavier and can be considered at rest during the ultrafast time scale of a laser pulse. Therefore, many electrons are pushed backward, effectively creating a dipole along the laser propagation direction [37]. The spatial separation of photoexcited electrons and ions leads to an ultrafast current surge, which radiates electric fields in the far-field region. Since the whole process is performed within a picosecond’s duration, the frequency of radiation is in the terahertz range [38]. As for the liquid with Ag NPs proposed in this work, the source of terahertz radiation is not only the ultrafast current surge mentioned above but also the coherent transition radiation, which is used to explain the radiation mechanism of liquid metal. When the electrons which are accelerated by the ponderomotive force pass through the metal–liquid interface, terahertz waves can be generated. In addition, Ag has much greater conductivity than liquid water [39,40], indicating that more carriers can be involved in the radiation process, leading to more intense terahertz emission.

The procedure of the chemical reduction method for PVA-stabilized Ag NP suspensions is presented in this study. By adjusting the dropping rate of reductant NaBH4, Ag NPs with quasispherical or chain-like nanostructures can be obtained. According to the experimental results, the radiant power at 0.5 THz from the quasispherical Ag NP suspension was five times stronger than that from liquid water in identical excitation conditions. In addition, the quasispherical Ag NPs remained stable when they were excited by laser pulses several times, and only a small decrease in the NP concentration was observed. The chain-like Ag NP suspension radiated more intense terahertz waves than that those from the quasispherical Ag NPs. However, it showed poor stability and changed significantly after laser excitation. We attribute this phenomenon to PVA damage caused by intense laser pulses. Therefore, Ag NPs with a chain-like structure cannot be recycled in a scenario of terahertz emission from a flowing liquid induced by femtosecond laser pulses.

## 5. Conclusions

In this work, an enhancement in terahertz radiation from a liquid with Ag NPs was demonstrated experimentally. The radiation power of 0.5 THz from the liquid with Ag NPs was five times stronger than that from liquid water in identical experimental conditions. Compared with chain-like Ag NP suspensions, quasispherical Ag NPs are more stable and can be used in a recyclable manner. This work proposes a liquid target for terahertz radiation which is embedded with metal nanoparticles, offering new possibilities for liquid terahertz sources. Further research is expected on the mechanism, enhancement, and control of these kinds of terahertz sources.

## Data Availability

The data presented in this study are available on request from the corresponding author.

## References

[1] Andreeva V.A., Kosareva O.G., Panov N.A., Shipilo D.E., Solyankin P.M., Esaulkov M.N., González de Alaiza Martínez P., Shkurinov A.P., Makarov V.A., Bergé L. (2016). Ultrabroad Terahertz Spectrum Generation from an Air-Based Filament Plasma. Phys. Rev. Lett..

[2] Zhang Y., Sun W., Wang X., Ye J., Feng S., Han P., Zhang Y. (2017). Active stabilization of terahertz waveforms radiated from a two-color air plasma. Opt. Lett..

[3] Buccheri F., Zhang X.C. (2015). Terahertz emission from laser-induced microplasma in ambient air. Optica.

[4] Ma D., Dong L., Zhang M., Wu T., Zhao Y., Zhang L., Zhang C. (2020). Enhancement of terahertz waves from two-color laser-field induced air plasma excited using a third-color femtosecond laser. Opt. Express.

[5] Ma D., Dong L., Zhang R., Zhang C., Zhao Y., Zhang L. (2021). Enhancement of terahertz wave emission from air plasma excited by harmonic three-color laser fields. Opt. Commun..

[6] Jin Q., Yiwen E., Zhang X.C. (2021). Terahertz aqueous photonics. Front. Optoelectron..

[7] Ponomareva E.A., Stumpf S.A., Tcypkin A.N., Kozlov S.A. (2019). Impact of laser-ionized liquid nonlinear characteristics on the efficiency of terahertz wave generation. Opt. Lett..

[8] Chen Y., He Y., Tian Z., Dai J. (2022). Lateral terahertz wave emission from laser induced plasma in liquid water line. Appl. Phys. Lett..

[9] Zhang L.L., Wang W.M., Wu T., Feng S.J., Kang K., Zhang C.L., Zhang X.C. (2019). Strong Terahertz Radiation from a Liquid-Water Line. Phys. Rev. Appl..

[10] Chen Y., He Y., Zhang Y., Tian Z., Dai J. (2021). Systematic investigation of terahertz wave generation from liquid water lines. Opt. Express.

[11] Dey I., Jana K., Fedorov V.Y., Koulouklidis A.D., Mondal A., Shaikh M., Kumar G.R. (2017). Highly efficient broadband terahertz generation from ultrashort laser filamentation in liquids. Nat. Commun..

[12] Jin Q., Yiwen E., Williams K., Dai J., Zhang X.C. (2017). Observation of broadband terahertz wave generation from liquid water. Appl. Phys. Lett..

[13] Jin Q., Dai J., Zhang X.C. (2018). Terahertz wave emission from a liquid water film under the excitation of asymmetric optical fields. Appl. Phys. Lett..

[14] Ponomareva E.A., Tcypkin A.N., Smirnov S.V., Putilin S.E., Yiwen E., Kozlov S.A., Zhang X.C. (2019). Double-pump technique—One step closer towards efficient liquid-based THz sources. Opt. Express.

[15] Jin Q., Zhang X.C. (2019). Enhancement of terahertz emission by a preformed plasma in liquid water. Appl. Phys. Lett..

[16] Sarpe C., Köhler J., Winkler T., Wollenhaupt M., Baumert T. (2012). Real-time observation of transient electron density in water irradiated with tailored femtosecond laser pulses. New J. Phys..

[17] Kim K.Y., Glownia J.H., Taylor A.J., Rodriguez G. (2007). Terahertz emission from ultrafast ionizing air in symmetry-broken laser fields. Opt. Express.

[18] Wang H.Y., Shen T. (2020). Unified theoretical model for both one- and two-color laser excitation of terahertz waves from a liquid. Appl. Phys. Lett..

[19] Ismagilov A.O., Ponomareva E.A., Zhukova M.O., Putilin S.E., Nasedkin B.A., Tcypkin A.N. (2021). Liquid jet-based broadband terahertz radiation source. Opt. Eng..

[20] Tcypkin A.N., Ponomareva E.A., Putilin S.E., Smirnov S.V., Shtumpf S.A., Melnik M.V., E Y., Kozlov S.A., Zhang X.C. (2019). Flat liquid jet as a highly efficient source of terahertz radiation. Opt. Express.

[21] Cao Y., Ling F., Zhang X.C. (2020). Flowing cryogenic liquid target for terahertz wave generation. AIP Adv..

[22] Balakin A.V., Coutaz J.L., Makarov V.A., Kotelnikov I.A., Peng Y., Solyankin P.M., Zhu Y., Shkurinov A.P. (2019). Terahertz Wave Generation from Liquid Nitrogen. Photonics Res..

[23] Chen Y., He Y., Liu L., Tian Z., Dai J. (2022). Scaling of the terahertz emission from liquid water lines by plasma reshaping. Opt. Lett..

[24] E Y., Zhang L., Tcypkin A., Kozlov S., Zhang C., Zhang X.-C. (2021). Broadband THz Sources from Gases to Liquids. Ultrafast Sci..

[25] E Y., Zhang L., Tcypkin A., Kozlov S., Zhang C., Zhang X.-C. (2022). Progress, challenges, and opportunities of terahertz emission from liquids. J. Opt. Soc. Am. B.

[26] Cao Y., Huang P., Zhang X.C. (2020). Broadband terahertz wave emission from liquid metal. Appl. Phys. Lett..

[27] Leemans W.P., Geddes C.G.R., Faure J., Tóth C., van Tilborg J., Schroeder C.B., Esarey E., Fubiani G., Auerbach D., Marcelis B. (2003). Observation of terahertz emission from a laser-plasma accelerated electron bunch crossing a plasma-vacuum boundary. Phys. Rev. Lett..

[28] Liao G., Li Y., Zhang Y., Liu H., Ge X., Yang S., Wei W., Yuan X., Deng Y., Zhu B. (2016). Demonstration of coherent terahertz transition radiation from relativistic laser-solid interactions. Phys. Rev. Lett..

[29] Solyankin P.M., Lakatosh B.V., Krivokorytov M.S., Tsygvintsev I.P., Sinko A.S., Kotelnikov I.A., Shkurinov A.P. (2020). Single Free-Falling Droplet of Liquid Metal as a Source of Directional Terahertz Radiation. Phys. Rev. Appl..

[30] Durán N., Durán M., De Jesus M.B., Seabra A.B., Fávaro W.J., Nakazato G. (2016). Silver nanoparticles: A new view on mechanistic aspects on antimicrobial activity. Nanomed. Nanotechnol. Biol. Med..

[31] Prabhu S., Poulose E.K. (2012). Silver nanoparticles: Mechanism of antimicrobial action, synthesis, medical applications, and toxicity effects. Int. Nano Lett..

[32] Abdul Kareem T., Anu Kaliani A. (2011). Synthesis and thermal study of octahedral silver nano-plates in polyvinyl alcohol (PVA). Arab. J. Chem..

[33] Kyrychenko A., Pasko D.A., Kalugin O.N. (2017). Poly (vinyl alcohol) as a water protecting agent for silver nanoparticles: The role of polymer size and structure. Phys. Chem. Chem. Phys..

[34] Navaladian S., Viswanathan B., Viswanath R.P., Varadarajan T.K. (2006). Thermal decomposition as route for silver nanoparticles. Nanoscale Res. Lett..

[35] Azad A.K., Dai J., Zhang W. (2006). Transmission properties of terahertz pulses through subwavelength double split-ring resonators. Opt. Lett..

[36] Hu J., Yang L., Zhu Y., Yang D.Q., Sacher E. (2020). Destabilization of PVA-stabilized Ag NPs color changes at low aqueous concentrations induced by aggregation and coalescence. Mater. Res. Express.

[37] Zhang X.-C., Buccheri F. (2018). Terahertz Photonics of Microplasma and beyond. Lith. J. Phys..

[38] Kim K.Y., Glownia J.H., Taylor A.J., Rodriguez G. (2012). High-Power Broadband Terahertz Generation via Two-Color Photoionization in Gases. IEEE J. Quantum Electron..

[39] Ageev I.M., Rybin Y.M. (2020). Features of Measuring the Electrical Conductivity of Distilled Water in Contact with Air. Meas. Tech..

[40] Bardeen J. (1940). Electrical conductivity of metals. J. Appl. Phys..

